# Ta-Doped Sb_2_Te Allows Ultrafast Phase-Change Memory with Excellent High-Temperature Operation Characteristics

**DOI:** 10.1007/s40820-020-00557-4

**Published:** 2021-01-04

**Authors:** Yuan Xue, Shuai Yan, Shilong Lv, Sannian Song, Zhitang Song

**Affiliations:** State Key Laboratory of Functional Materials for Informatics, Shanghai Institute of Microsystem and Information Technology, Chinese Academy of Sciences, Shanghai, 200050 People’s Republic of China

**Keywords:** Phase-change memory, High speed, Ta, High-temperature operation

## Abstract

Phase-change memory (PCM) has considerable promise for new applications based on von Neumann and emerging neuromorphic computing systems. However, a key challenge in harnessing the advantages of PCM devices is achieving high-speed operation of these devices at elevated temperatures, which is critical for the efficient processing and reliable storage of data at full capacity. Herein, we report a novel PCM device based on Ta-doped antimony telluride (Sb_2_Te), which exhibits both high-speed characteristics and excellent high-temperature characteristics, with an operation speed of 2 ns, endurance of > 10^6^ cycles, and reversible switching at 140 °C. The high coordination number of Ta and the strong bonds between Ta and Sb/Te atoms contribute to the robustness of the amorphous structure, which improves the thermal stability. Furthermore, the small grains in the three-dimensional limit lead to an increased energy efficiency and a reduced risk of layer segregation, reducing the power consumption and improving the long-term endurance. Our findings for this new Ta–Sb_2_Te material system can facilitate the development of PCMs with improved performance and novel applications.

**Electronic supplementary material:**

The online version of this article (10.1007/s40820-020-00557-4) contains supplementary material, which is available to authorized users.

## Highlights

- Phase-change memory based on Ta-doped antimony telluride (Sb_2_Te) exhibits both high-speed characteristics and excellent high-temperature characteristics, allowing improved performance and new applications.
- The high coordination number of Ta and the strong bonds between Ta and Sb/Te atoms enhance the robustness of the amorphous structure, ensuring good thermal stability.
- Through the three-dimensional limit, the formation of small grains reduces the power consumption and improves the long-term endurance.

**Electronic supplementary material:**

The online version of this article (10.1007/s40820-020-00557-4) contains supplementary material, which is available to authorized users.

## Graphical abstract

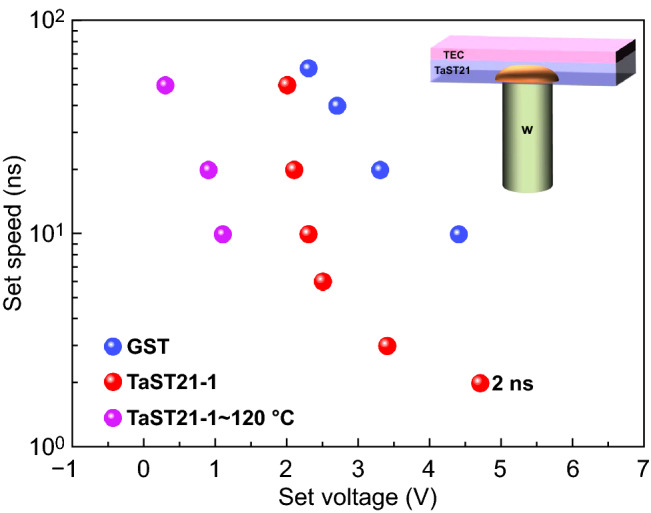

## Introduction

In the current era of information explosion, the demand for faster data access and higher-capacity storage has increased, making traditional data storage devices inadequate [1, 2]. Phase-change memory (PCM) provides a promising solution to this problem, and it has been commercialized as the 3D XPoint product from Intel [3, 4]. PCM, in which digital information is encoded by phase-change materials with large contrasts in electrical resistance or optical reflectivity depending on the crystallinity, has recently attracted significant attention owing to its remarkable properties, e.g., its logic compatibility, low fabrication costs, excellent scalability, and high endurance [5–8]. Recently, it has been regarded as storage-class memory and artificial synapses to fill the performance gap between flash memory and dynamic random-access memory (DRAM) in von Neumann systems and emerging neuromorphic systems [9–12]. However, this places greater requirements on the PCM. In automotive applications, high-speed and elevated-temperature operation is needed to process large amounts of data efficiently and ensure normal storage at high working temperatures [13].

The performance metrics of the PCM, including the operation speed, thermal stability, and power consumption, are closely related to the properties of the phase-change material and the device structure. The phase-change material is compatible with the existing manufacturing process; therefore, optimizing the properties of the phase-change material is an effective method for improving the device performance. In a PCM, to perform a Reset operation, an electrical pulse of a large amplitude is employed to enable melt–quench of the material, thereby switching the material to the high-resistance amorphous state [14]. Conversely, in a Set operation, the material is heated between the crystallization temperature and the melting point (*T*_m_) by a longer pulse, which switches the material to the low-resistance crystalline state [15]. The thermal stability of the amorphous state corresponds to the integrity of the stored data. The property is represented by the crystallization temperature (*T*_c_); a higher crystallization temperature corresponds to a more stable amorphous state. The data are erased by heating the amorphous state with a pulse, and a higher crystallization speed corresponds to a shorter pulse width. A material with a higher melting temperature requires a higher operating energy for the Reset operation. Hence, developing ideal phase-change materials with high crystallization speed, good crystallization temperature, and low melting temperature is critical for achieving excellent performance in applications in challenging scenarios [16, 17].

Ge_2_Sb_2_Te_5_ (GST) has been widely used as the core material in PCM products. However, its low crystallization temperature and large grains result in a slow Set process and poor thermal stability, which is the bottleneck for applying PCM in many challenging scenarios [18–20]. Owing to its growth-dominated crystallization mechanism and the presence of multiple Sb_2_ layers in its crystal structure, the δ-phase Sb–Te binary compound is a high-speed phase-change material. However, its low thermal stability makes it unsuitable for many applications [21, 22]. In previous studies, a series of modifications were adopted, e.g., using C [23], Y [24], Cu [25], Zr [26], and Al [27], most of which enhanced the thermal stability but reduced the operation speed. Additionally, owing to the weaker grain-growth restriction, large grains are formed after long-term operation, raising concerns regarding the reliability of these devices.

Thus, it is a significant challenge to exploit the PCM technology for high-performance applications. Ta has a large atomic mass and is difficult to move; thus, when it is introduced into a phase-change material, it can prevent the growth of the crystal grains and thus aid in improving the thermal stability, operation speed, and endurance of the PCM [28, 29]. In this study, Sb_2_Te was doped with a Ta alloy for improving the structural rigidity of the amorphous phase to achieve excellent thermal stability and enhance the operation speed. A systematic experimental investigation of the Ta-doped Sb_2_Te alloy was conducted in which the device performance was evaluated. Moreover, the device characteristics at an elevated temperature were examined, and the results indicated that these PCMs have robust device performance in a high-temperature working environment. Finally, the mechanism underlying the outstanding device performance was discussed.

## Experimental Section

Sb_2_Te and Ta-Sb_2_Te films were fabricated using Ta and Sb_2_Te targets, whose stoichiometric ratios were determined via energy-dispersive X-ray spectroscopy. Films with a thickness of 100 nm were deposited on SiO_2_ and Si substrates for resistance–temperature (*R*–*T*) measurements, X-ray diffraction (XRD) spectroscopy, and Raman spectroscopy. The structural evolution during transformation was investigated via in situ transmission electron microscopy (TEM) using a 20-nm-thick film deposited on an ultrathin C film supported by Cu grids. Finally, electrical measurements were performed on the T-shaped PCM with a 190-nm-diameter bottom electrode. A 100-nm-thick TaST21-1 film and a 10-nm-thick TiN film were deposited on the W bottom electrode, as an adhesion layer. A 300-nm-thick Al film served as the top electrode. Electrical tests were performed using a Keithley 2400C source meter, a Tektronix AWG5002B pulse generator, and a Picosecond Pulse Labs Model 10070A pulse generator.

The calculations were performed within the framework of the density functional theory (DFT) as implemented in the Vienna Ab initio simulation package code. The electron–ion interaction was described by the projector augmented wave (PAW) pseudopotentials, and the PAW potentials were used with the generalized gradient approximations of the Perdew–Burke–Ernzerhof exchange–correlation function [30]. We obtained the structure of the amorphous system using 225-atom models of TaST21 (8 Ta, 142 Sb, 75 Te) and ST21 (150 Sb, 75 Te) via ab initio molecular dynamics (AIMD) simulation. Each step was set as 3 fs. To obtain an amorphous structure, the model was melted and equilibrated at 3000 K for 9 ps, quenched to 1200 K for 30 ps, equilibrated at 1200 K for 30 ps, quenched to 300 K for 60 ps, and finally maintained at 300 K for 15 ps. The structure was annealed at 600 K for 30 ps to calculate the mean-squared displacement (MSD).

## Results and Discussion

### Thermodynamic Properties and Microstructure

**Fig. 1:**
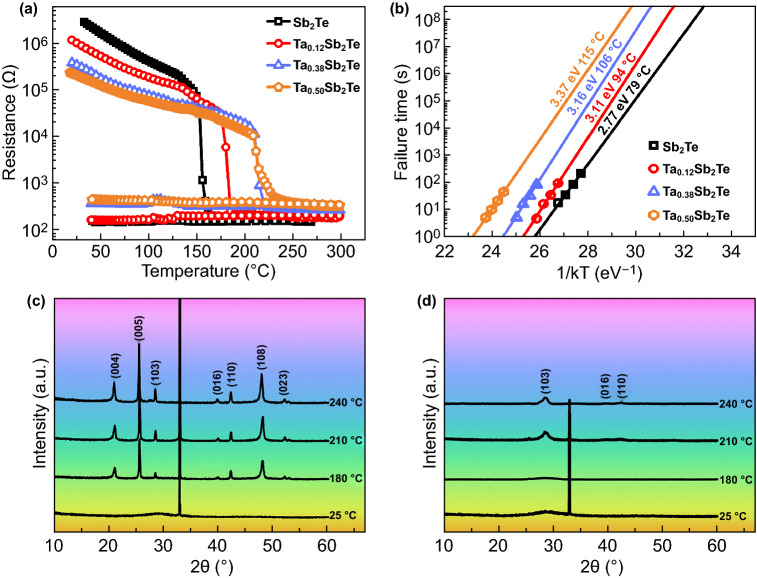
**a** In situ R-T measurement results for ST21 and Ta-doped components. **b** Failure time versus the reciprocal of the temperature. XRD curves of **c** ST21 and **d** TaST21-1 films at different temperatures

The thermal stability of the amorphous state, which is vital in nonvolatile PCM data storage, was characterized via in situ *R*-*T* measurements. Figure 1a shows the *R*-*T* measurement results for Sb_2_Te and TaST21 films at a heating rate of 20 °C min^−1^ and a cooling rate of 100 °C min^−1^. The precipitous drop point of the *R*-*T* curves indicates the crystallization temperature (*T*_c_) from the amorphous state to the crystalline state. The *T*_c_ values of ST21, TaST21-1, TaST21-2, and TaST21-3 were 164, 183, 197, and 209 °C, respectively. The higher *T*_c_ relative to GST was due to the Ta doping, which improved the thermal stability of the amorphous state [31]. The crystalline-state resistance increased with the Ta content, allowing efficient Joule heating in the process of phase transformation, which is advantageous for reducing the power consumption [32]. As shown in Fig. 1b, the activation energy of crystallization and the temperature for 10-year data retention (*T*_10-year_) were calculated using the Arrhenius equation [33]. The maximum failure temperatures after 10 years for the TaST21-1, TaST21-2, and TaST21-3 films were determined to be 94, 106, and 115 °C, respectively, indicating the more stable amorphous state. The increased activation energy (*E*_a_) exhibited better thermal stability with a larger energy barrier.

The lattice information of ST21 and TaST21-1 was obtained via XRD measurements, as shown in Fig. 1c and d. All the samples were annealed in N_2_ for 3 min. The absence of diffraction peaks of ST21 and TaST21-1 confirms that the deposited film was in an amorphous state. Diffraction patterns of the hexagonal lattice appeared for ST21 when the annealing temperature reached 180 °C. For TaST21-1, the diffraction peak did not appear until the temperature reached 210 °C, similar to the case of hexagonal ST21. No phase separation occurred until the temperature increased to 240 °C, indicating good uniformity.

**Fig. 2:**
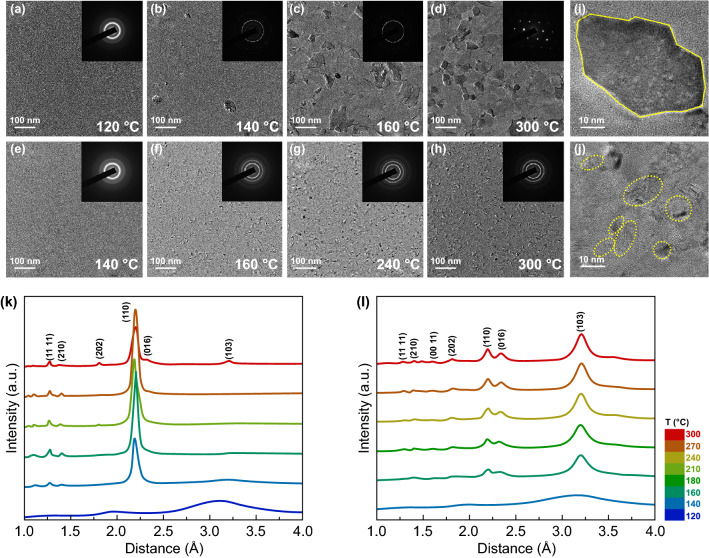
BF TEM and SEAD patterns of the **a**–**d** ST21 film and **e**–**f** TaST21-1 film during in situ heating, with a heating rate of 10 °C min^−1^. Insets: the corresponding SAED patterns of ST21 and TaST21-1 obtained via in situ TEM. **i, j** HRTEM images of the two aforementioned samples. The raw radially integrated diffraction curves of the electronic diffraction intensity of ST21 and TaST21-1 are presented in (**k**) and (**l**)

Understanding the structural evolution that occurs during transformation is of fundamental importance. Figure 2a–d and e–h presents TEM bright-field (BF) images of the ST21 and TaST21-1 films, respectively. The crystallization of the ST21 film started at 140 °C, as indicated by the formation of a few small crystal nuclei with diverse shapes. As the temperature increased, a typical growth-dominated crystallization characteristic with a preference for the formation of larger grains was observed. The grain size reached 100 nm at 160 °C. During the subsequent heating process, the grain size continued to increase, reaching several hundred nanometers, and the polycrystalline rings of the corresponding selected-area electron diffraction (SAED) patterns exhibited discontinuities. Large grains led to poor adhesion between the phase-change film and the substrate owing to the high probability of grain-boundary diffusion or sliding, which was disadvantageous for the reliability of the PCM. They also had a significant negative impact on the device performance, particularly the operation speed and power consumption. In the case of Ta-ST21, the crystallization temperature (160 °C) increased with the formation of small crystal nuclei. In contrast to the case of ST21, as the temperature increased, the crystal grains did not grow significantly, but new crystal nuclei were continuously formed. Even when the temperature increased to 300 °C, as shown in Fig. 2h, the diffraction ring had good continuity, and crystalline grains were distributed homogenously under this annealing treatment. It is evident that a small amount of Ta dopant is sufficient to suppress the grain growth effectively, play the role of the three-dimensional limit, and change the crystallization behavior from growth-dominated to nucleation-dominated. As shown in Fig. 2i and j, the grain growth for ST21 and TaST21-1 was analyzed using high-resolution TEM (HRTEM) images. Oversized grains (> 60 nm) were observed in the ST21 film, with clear grain boundaries. For the doped sample, the grains size was significantly reduced (to approximately 10 nm). Assuming that the grains were perfectly spherical, the volume was reduced by a factor of 226. The grain-size reduction increased the number of grain boundaries, providing phonon and electron scattering centers, which was highly desirable for reducing the heat dissipation and transport in the programming regions, thus increasing the memory density and reducing the power consumption. Figure S1 presents the density change between the amorphous and crystalline states, which was evaluated via the X-ray reflectivity technique. As shown, the doped material exhibited a reduced density change, which was even smaller than that of the GST (6.5%), indicating that the inhibition of the grain growth had a distinct effect. Additionally, the raw radially integrated diffraction curves of the electronic diffraction intensity were extracted from the SAED patterns, as shown in Fig. 2k and l [34]. The TaST21-1 film can be indexed as a hexagonal Sb_2_Te structure. No extra peak appeared for the film, indicating that the film had a single phase without phase separation, which suggests that the Ta doping significantly affected the crystallization behavior without forming a new structure. For the TaST21-1 film, the intensities of the diffraction peaks did not change with the increasing temperature, in agreement with the TEM BF images.

**Fig. 3:**
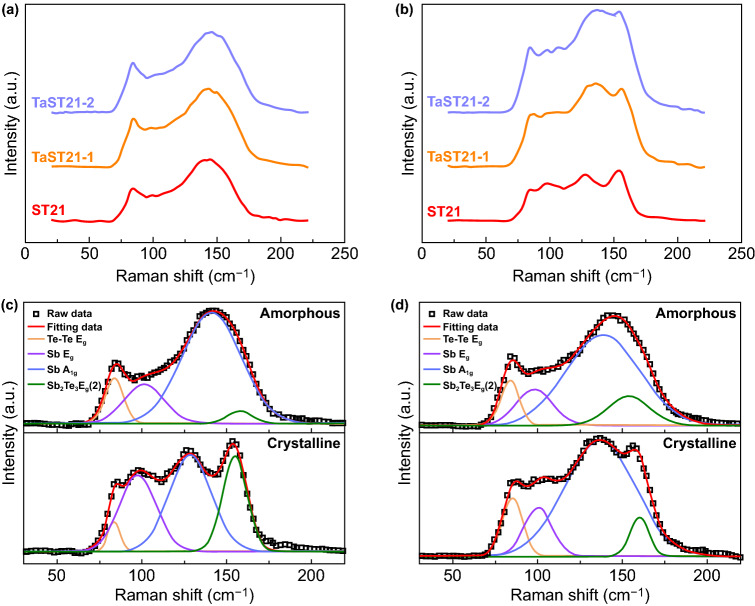
Raman spectroscopy results for the ST21, TaST21-1, and TaST21-2 films in the **a** amorphous state and **b** crystalline state. Gaussian fitting of the Raman spectroscopy data for crystalline and amorphous **c** ST21 and **d** TaST21-1 films

For elucidating the effect of Ta on the original bonding environment changes, Raman spectroscopy was performed to identify the unique vibration modes of the bonds between Ta, Sb, and Te atoms. The curves for amorphous ST21 and TaST21 were similar, as shown in Fig. 3a. They contained an increased bandwidth covering the frequency range of 70–170 cm^−1^ without obvious vibration peaks due to the complex binding environment in the amorphous state. For crystalline ST21, because of the long-range order, an obvious vibration peak appeared. The initial structure of ST21 consisted of nine layers of atoms along the *c* axis, including five layers of Sb_2_Te_3_ and four layers of Sb atoms. However, after the Ta doping, the phenomenon of peak widening occurred in the high-frequency band, indicating that the Ta disturbed the crystal lattice. To better distinguish the different vibration modes, Gaussian line-fitting was applied to the Raman results of ST21and TaST21-1, as shown in Fig. 3c and d. The detailed parameters obtained after the fitting are presented in Table 1. There were only four vibration modes for both components. Peaks A and D were attributed to the *E*_*g*_ mode of Te–Te bonds and the $A_{{1{\text{g}}}}^{2}$ mode of Sb_2_Te_3_, respectively [35, 36]. Peaks B and C were derived from the *E*_*g*_ and *A*_*1g*_ modes of Sb–Sb bonds, respectively. Vibration modes with higher peak intensities dominated. Among the peaks, the C peak had the highest intensity, indicating that the Sb–Sb homogenous bond prevailed in ST21 and TaST21. The intensity of the Sb–Sb homogeneous bond was significantly higher in TaST21 than in ST21. It is inferred that Ta is more inclined to combine with Te, forming more Sb–Sb bonds, which is desirable for the realization of high-speed PCMs. Additionally, the intensity ratio of the D peak to the C peak was lower for TaST21 than for ST21, indicating that the ratio of Sb_2_Te_3_ was significantly lower for TaST21 than for ST21. This may have been due to the suppression of the formation of Sb_2_Te_3_ in Sb_4_-(Sb_2_Te_3_) unit cells after the Ta doping [37].

**Table 1:** Peak identity of Raman spectra of as-deposited and crystalline ST21, TaST21-1 film

Peak identity	Amorphous		Crystalline	
	Intensity (arb. units)	Wavenumber (cm^−1^)	Intensity (arb. units)	Wavenumber (cm^−1^)
*A*	293.37 (224.72)	83.4 (83.74)	375.66 (125.98)	84.83 (83.56)
*B*	253.1 (195.15)	97.87 (101.24)	319.97 (319.60)	100.1 (97.45)
*C*	589.15 (554.31)	138.32 (141.23)	757.93 (406.95)	136.55 (128.71)
*D*	190.43 (65.01)	153.34 (157.7)	255.23 (401.73)	159.65 (154.93)

Data for ST21 are shown in parentheses

### Device Performance

**Fig. 4:**
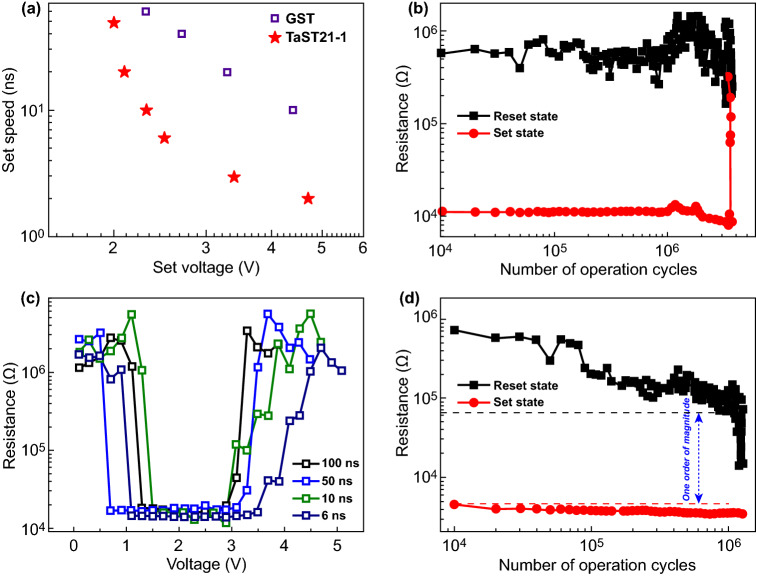
**a** Comparison of the Set speeds. **b** Endurance of the TaST21-1 device. **c, d** Device performance of TaST21-1 at 100 °C

The electrical properties of the memory cell based on the TaST21-1 alloy were examined. First, the effect of the Ta dopant on the Set process was investigated. For a direct comparison, measurement data for a GST-based cell of the same size are also presented. As shown in Fig. 4a, the time of the applied voltage pulses varied from 50 to 2 ns, and the magnitude of the voltage varied from 2 to 4.7 V. A higher applied voltage corresponded to a higher Set speed of the device. Clearly, TaST21-1 had a faster Set process than the GST device, which required 10 ns, thus reaching the standards for replacing DRAM. Figure 4b shows the endurance performance of the cell. The number of cycles reached 3.6 × 10^6^, which was far superior to that for the ST21 device. The superior endurance largely originated from the uniform structure, minimal change in density, and small grain size. Moreover, to predict the overall distribution of the Reset and Set resistance, a 14-bit PCM array was tested with different pulse durations (Figs. S2 and S3). The slope of the distribution function reflected the concentration of the resistance distribution of the Set and Reset states. A larger slope corresponded to a more concentrated resistance distribution. The optimal operating conditions of the device were 1.9 V for Set and 4.9 V for Reset under a pulse width of 100 ns. There was a window of 1.6 orders of magnitude between the highest resistance of the Set state and the lowest resistance of the Reset state. Similarly, a resistance ratio of 1.4 was maintained under the conditions of 2.3 V/6 ns and 5.5 V/6 ns, ensuring a reliable and adjustable logical partition. The resistance distribution curve was approximately a straight line, indicating that the resistance of the 14-bit array can be regarded as a normal distribution. The foregoing experiments confirm the excellent uniformity and repeatability of the cell resistance. The TaST21-1 alloy appears to be a suitable candidate for fast-switching and high-endurance PCM applications. As the dimensions are reduced (accompanied by a sharply reduced program energy), the lifetime of TaST21-1 based cells will increase exponentially, indicating considerable potential to satisfy the stringent requirements of DRAM-like applications.

Additionally, normal Set and Reset operations at high temperatures are critical for PCMs. We performed the operation of TaST21-1 cells at different ambient temperatures. The temperature was increased from room temperature in steps of 20 °C until it reached 140 °C. The performance at 100 °C is shown in Fig. 4c, d, and the performance at the remaining temperatures is shown in Fig. S4. Despite the temperature increasing to 100 °C, the cell readily achieved complete Set and Reset operations with a 6-ns pulse width, satisfying the desired performance of high-speed devices. The *U*_Set_ and *U*_Reset_ were lower at 120 °C than at 40 °C, because less energy was required to reset at an elevated temperature. Furthermore, the endurance capability was maintained for > 10^5^ cycles at 120 °C. The TaST21-1 device successfully operated at 120 °C, according to the measurement data. Such devices are also beneficial for solving thermal-crosstalk issues, and the operation capability at 120 °C is important in automobile electronics.

Phase-change device cells operate via Joule heating induced by electrical pulses. However, only a small fraction of the Joule heating is used for the phase transition, and most of the heat diffuses into the surrounding environment. Hence, using a material with a low thermal conductivity is advantageous for enhancing the energy efficiency. To directly explain the effect of the thermal conductivity on the power consumption, a two-dimensional finite analysis was performed in which the temperature distributions of the cells during the Reset operation at 25 and 120 °C were simulated, as shown in Fig. S5. Clearly, the highest temperature was located in the middle of the mushroom-shaped temperature distribution, and the heat generated was mainly concentrated in the active phase-change area on the bottom electrode contact. At the same ambient temperature, the peak temperature of TaST21-1 was significantly higher than the peak temperature of ST21, indicating that the Ta doping reduced the heat dissipation; thus, most of the Joule heating was used for inducing the phase change. Therefore, TaST21-1 has a high thermal efficiency and a low power consumption. Additionally, the peak temperature in the central region was significantly higher at 120 °C than at 25 °C. This indicates that at a higher ambient temperature, a lower pulse voltage is needed to generate sufficient energy to crystallize the phase-change material compared with devices operating at room temperature. This explains why the devices require less power and lower operating voltages at higher temperatures.

**Fig. 5:**
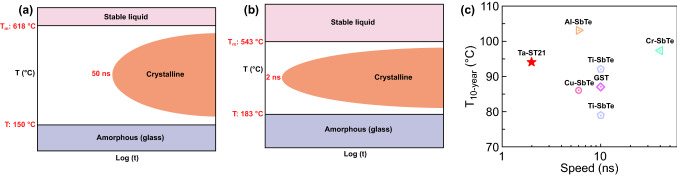
**a**, **b** Schematic plot of GST and TaST21 about *T*_*c*_, *T*_*m*_, and the operation speed. **c** Comparison curves of the speed and data retention for doped Sb–Te systems

A comparison of *T*_c_, *T*_m_, and the crystallization speed between GST and TaST21 is shown in Fig. 5 [38]. Compared with GST, TaST21 exhibited higher stability of the amorphous phase, a lower melting point, and a higher speed. A comparison curve of the speed and data retention based on a typical doped Sb–Te system is shown in Fig. 5c. Although some doped alloys can achieve higher data retention than TaST21, e.g., Al-SbTe and Cr-SbTe, only TaST21 has a speed of < 5 ns. Thus, the developed TaST21 phase-change material has excellent performance at elevated temperatures for automobile electronics.

### Amorphous Structure

**Fig. 6:**
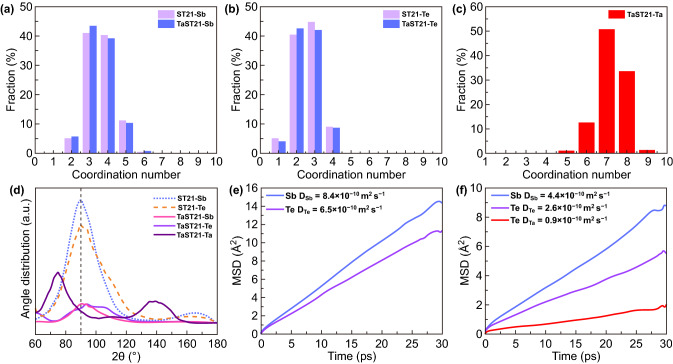
**a**–**c** Coordination-number distributions of Sb, Te, and Ta in amorphous ST21 and TaST21. **d** Bond-angle distributions around Sb, Te, and Ta in two models. **e, f** MSD curves for each atom in amorphous ST21 and TaST21 at 600 K for 30 ps

The structural properties of amorphous chalcogenides significantly affect their crystallization properties. Thus, an AIMD simulation was conducted to examine the amorphous structure of TaST21, as shown in Fig. 6, with a cutoff of 3.1 Å for all atomic pairs. Figure 6a shows the distribution of the coordination numbers for Ta, Sb, and Te atoms in ST21 and TaST21. For ST21, the majority of Sb atoms had threefold or fourfold coordination. Additionally, the Te atoms mainly had twofold or threefold coordination. The Ta did not affect the main coordination number of Sb or Te, indicating that the coordination environment was similar between ST21 and TaST21. The large peak position of the bond-angle distribution remained around 90°, indicating that most atoms were in a defective octahedral-like environment. Thus, there was no significant change in the local environment of Sb and Te. For Ta atoms, the coordination number is relatively high (mainly sevenfold coordination). A high coordination number is beneficial for enhancing the rigidity of atomic matrices and thereby improving the thermal stability of the amorphous material.

The charge-density difference was used to characterize the bonding between cations and anions in the amorphous state, as shown in Fig. S7. The incorporation of Ta increased the strength between Ta and Sb/Te, thereby improving the structural stability of TaST21; this ensured a high viscosity of the amorphous structure and better thermal stability of the amorphous state. The MSDs of ST21 and TaST21 are shown in Fig. 6e and f. The diffusion coefficient was extracted from the slopes of the curves. Notably, the diffusion coefficients of Sb and Te decreased sharply owing to the Ta dopant, confirming that the Ta atoms played a critical role in the TaST21 model. In general, the diffusion coefficient is inversely proportional to the activation energy of the amorphous state. Thus, Ta atoms increase the thermal stability of the amorphous material, resulting in a higher crystallization temperature relative to ST21. Moreover, for the overall system, the diffusion coefficient of Te atoms is reduced to a greater extent than that of Sb atoms, which is beneficial to the stability of the network structure.

## Conclusion

Detailed characterizations of the film properties and measurements of the device performance indicated that Ta-modified Sb_2_Te has considerable potential for PCM, combining the attractive characteristics of an ultrahigh speed and excellent performance at elevated temperatures. According to the results of Raman spectroscopy and an AIMD simulation, Ta prefers to bond with Te, inevitably increasing the atomic binding strength of the structure, which is a major reason for the high thermal stability. Additionally, the increase in Sb–Sb homogeneous bonds results in an ultrahigh speed. The grains are confined to smaller sizes owing to the three-dimensional limit of the Ta dopant, leading to a high energy efficiency for Joule heating, a reduced risk of stress and segregation, a low power consumption, good endurance of 10^6^ cycles, and high reliability. The effect of Ta on the microstructure of Sb_2_Te was the key to its excellent properties, and Ta-modified Sb_2_Te is a promising material for PCM applications.

## Electronic supplementary material

Below is the link to the electronic supplementary material.

Supplementary material 1 (PDF 692 kb)
