# Supplementary material for: Ta-Doped Sb2Te Allows Ultrafast Phase-Change Memory with Excellent High-Temperature Operation Characteristics
Source: Nanomicro Lett. 2021 Jan 4;13:33. doi: 10.1007/s40820-020-00557-4 (PMC8187703; doi:10.1007/s40820-020-00557-4)
Supplement: Supplementary file 1 — Supplementary material 1 (PDF 692 kb) [file 40820_2020_557_MOESM1_ESM.pdf]

Supporting Information for

Ta-Doped Sb2Te Allows Ultrafast Phase-Change Memory with Excellent High-Temperature Operation Characteristics

Yuan Xue[1], Shuai Yan[1], Shilong Lv[1], Sannian Song[1,*], Zhitang Song[1,*]

[1]State Key Laboratory of Functional Materials for Informatics, Shanghai Institute of Microsystem and Information Technology, Chinese Academy of Sciences, Shanghai 200050, People's Republic of China

*Corresponding authors. E-mail: songsannian@mail.sim.ac.cn (S. Song), ztsong@mail.sim.ac.cn (Z. Song)

Supplementary Figures

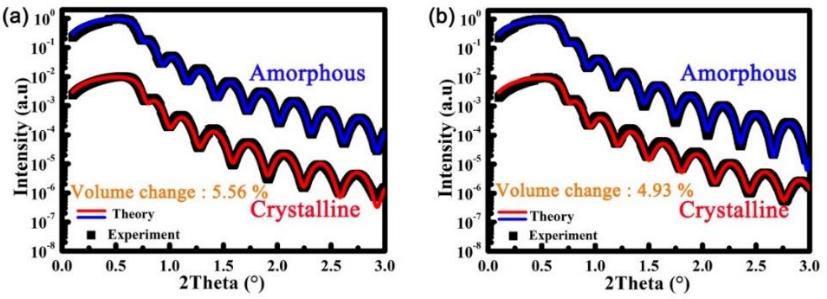

Fig. S1 X-ray reflectivity (XRR) measurement results and fitting, for (**a**) ST21 (**b**) TaST21-1. Black lines indicate the original data, red lines indicate the fitting results of the amorphous state, and blue lines indicate the fitting results of the crystalline state

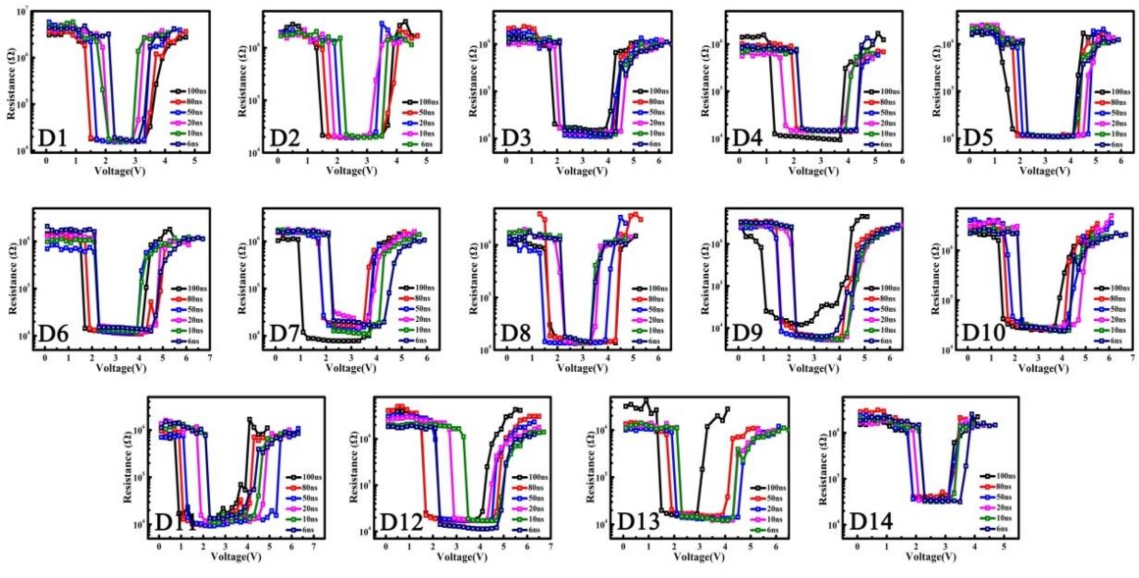

Fig. S2 Performance of devices in a 14-bit array based on TaST21-1

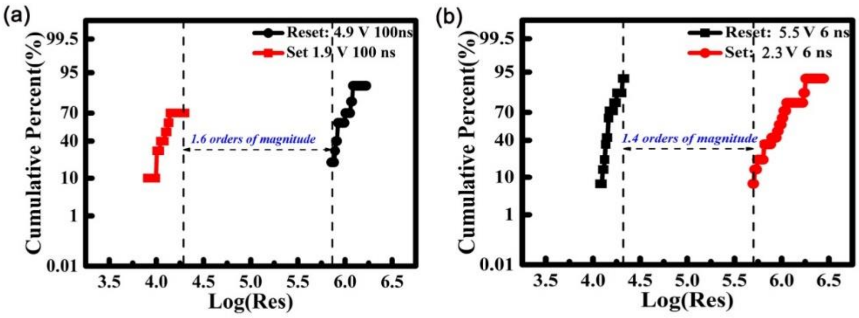

Fig. S3 Cumulative distribution of Set and Reset resistance of devices in the 14-bit array based on TaST21-1. (**a**) The measurement results when the pulse width is fixed at 100 ns. The optimal condition of Set and Reset operation is 1.9 V and 4.9 V, respectively. (**b**) The measurement results when the pulse width is fixed at 6 ns. The optimal condition of Set and Reset operation is 2.3 and 5.5 V, respectively

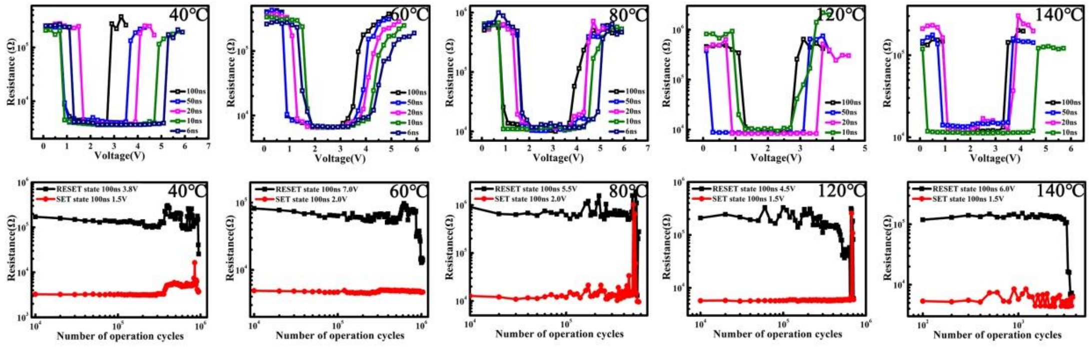

Fig. S4 Performance of TaST21-1 based phase change memory at different ambient temperatures

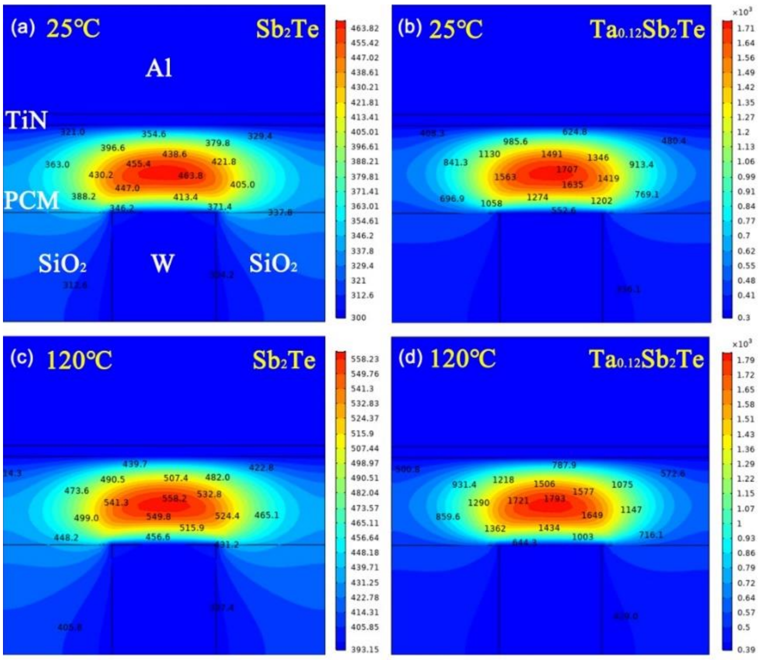

Fig. S5 Temperature distribution of a phase change memory based on ST21 and TaST21-1 at different ambient temperatures

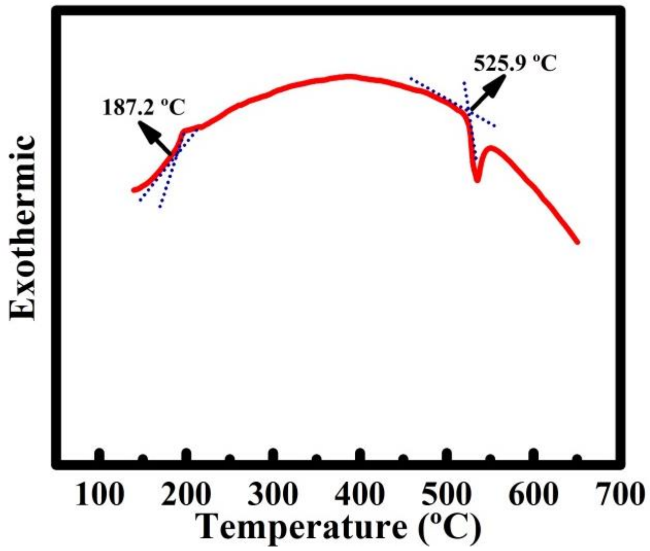

Fig. S6 Differential scanning calorimetry (DSC) measurement of a TaST21-1 film. The heating rate is 10 °C min^{-1}

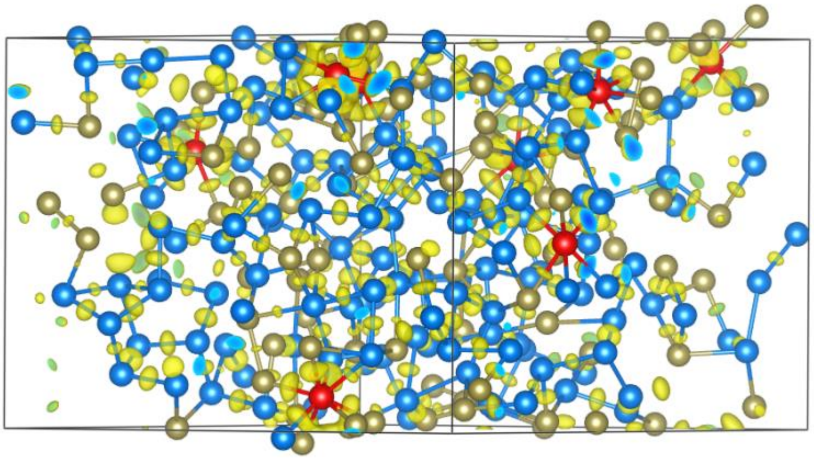

Fig. S7 Structure model of a-TaST21 with CDD
